# Therapeutic HL-Contact Lens versus Standard Bandage Contact Lens for Corneal Edema: A Prospective, Multicenter, Randomized, Crossover Study

**DOI:** 10.1155/2020/8410920

**Published:** 2020-09-21

**Authors:** Ofer Daphna, Michael Mimouni, Yariv Keshet, Meydan Ben Ishai, Irina S. Barequet, Boris Knyazer, Ewa Mrukwa-Kominek, Tomasz Zarnowski, Malca Chen-Zion, Arie Marcovich

**Affiliations:** ^1^EyeYon Medical, Ness Tziona, Israel; ^2^Department of Ophthalmology, Rambam Health Care Campus, Affiliated to the Bruce and Ruth Rappaport Faculty of Medicine, Technion-Israel Institute of Technology, Haifa, Israel; ^3^Department of Ophthalmology, Rabin Medical Center, Petach Tikva, Israel; ^4^Goldschleger Eye Institute, Sheba Medical Center, Tel Hashomer, Affiliated to the Sackler Faculty of Medicine, Tel-Aviv University, Tel-Aviv, Israel; ^5^Department of Ophthalmology, Soroka University Medical Center and Faculty of Health Sciences, Ben-Gurion University of the Negev, Beer Sheva, Israel; ^6^Department of Ophthalmology, Silesian University of Medicine, Katowice, Poland; ^7^Department of Diagnostics and Microsurgery of Glaucoma, Medical University of Lublin, Lublin, Poland; ^8^Department of Ophthalmology, Kaplan Medical Center and the Weizmann Institute of Science, Rehovot, Israel

## Abstract

**Introduction:**

To compare the safety and efficacy of the Therapeutic Hyper-CL™ lens versus a standard bandage contact lens (PureVision B&L) for chronic corneal edema.

**Methods:**

Prospective, multicenter, randomized, crossover study. Chronic corneal edema patients were randomized to one of two arms. The first arm was fitted with the Therapeutic Hyper-CL™ lens while the second arm was fitted with a standard soft bandage contact lens. Both arms were treated with 5% sodium chloride 6 times a day. After a 7-day treatment period, there was a 7-day washout period, after which the arms were crossed over. Patients were evaluated at days 0 (baseline), 7 (following first treatment allocation), 14 (following washout), and 21 (following second treatment allocation). The primary outcomes were 3 lines of BCVA (best corrected visual acuity) improvement.

**Results:**

In total, 49 patients were enrolled. There was significantly greater BCVA improvement rate >3 lines (30.4% versus 17.4%, *P*=0.04) in the Therapeutic Hyper-CL™ lens group. The mean change in BCVA lines was significantly greater for the Therapeutic Hyper-CL™ lens (3.4 ± 6.7 versus 0.9 ± 2.3, *P*=0.02).

**Conclusions:**

The Therapeutic Hyper-CL™ lens was associated with a higher chance for significant visual acuity improvement when compared to a standard bandage contact lens combined with 5% sodium chloride. This trial is registered with NCT02660151.

## 1. Introduction

Corneal edema, and associated loss of visual acuity, affects millions worldwide and is one of the most common complications of cataract surgery [1]. Excessive corneal hydration is largely the result of endothelial cell dysfunction or deficiency, which can arise following ocular surgery or trauma or can be of congenital, degenerative, metabolic, neoplastic, dystrophic, or inflammatory origin [2]. Other causes for corneal edema may include corneal scarring, corneal inflammation, corneal infection, and corneal dystrophies. This is counteracted externally, by the overlying epithelium, which forms a passive barrier to flow of water and electrolytes into the cornea and internally, by the endothelium. Regarding the epithelium part in this mechanism, papers and theories were studied and published including the use of basic fibroblast growth factor or the use of cysteine or epithelium-derived IL-33 mast cells following corneal injuries [3–5].

The collagen-rich stromal layer, constituting about 90% of the corneal tissue, features highly polar interfibrillar glycosaminoglycans, which exert a swelling pressure, driving imbibition of fluid from the anterior chamber into the cornea [6].

The endothelium maintains the cornea in a dehydrated state, by actively pumping water out into the aqueous [7]. In parallel, water evaporation via the corneal tear film yields a slightly hypertonic solution at the eye surface, which then draws fluid out of the cornea by way of osmosis. Overall, any imbalance in the forces regulating hydration homeostasis may result in increased fluid accumulation and corneal swelling. Other causes of endothelial disfunction may include inflammation or trauma but this paper will not discuss those issues furthermore.

Conservative management approaches are taken in cases of transient corneal edema and as a bridge to transplantation, which is often delayed due to corneal graft tissue shortage [8]. These include mainly hypertonic agents, alongside adjuvant bandage contact lenses, which temporarily alleviate pain and discomfort [9]. While hypertonic solutions effectively draw fluid from the stroma, they suffer from a short retention time and subsequently limited effect, largely due to their elimination via reflex tears and blinking [10].

Therapeutic Hyper-CL™ (EyeYon Medical, Ness Tziona, Israel) is a novel contact lens designed to increase the contact time of eye drops on the corneal surface. Its innovative design includes dual base curves, fenestrations, and a reservoir. This design forms a cavity between the lens and the cornea, in which the instilled eye drops become trapped, extending their contact time with the cornea. The fenestrations improve drug accessibility to the epicorneal space, as well as tear mixing and consequential oxygen supply to the cornea.

The purpose of this multicenter, prospective, randomized, crossover study was to assess and compare the safety and efficacy of the Therapeutic Hyper-CL™ versus a standard bandage contact lens in management of corneal edema with secondary visual impairment.

## 2. Methods

The study protocol was approved by the ethics committee of each site and was executed in accordance with the International Conference of Harmonization Good Clinical Practice guidelines and the Declaration of Helsinki. The trial was registered in ClinicalTrials.gov (Identifier: NCT02660151) and conducted in four medical centers in Israel (Soroka Medical Center and Sheba Medical Center) and Poland (Katowice Medical Center and Lublin Medical Center).

All patients provided signed, informed consent before initiation of any study procedures.

### 2.1. Patients

Adult patients suffering from chronic corneal edema for >3 months and with impaired BCVA (≤6/20 Snellen) clinically demonstrated as secondary to corneal edema were eligible to participate in the study. Patients with active herpes keratitis, presenting a severely scarred, eroded, or infected cornea, or with glaucoma shunts and/or a bleb were excluded.

### 2.2. Study Design

Patients were equally allocated (with a 1 : 1 ratio) to one of the following two crossover regimens based on a randomization scheme with blocks stratified by center: A-B, B-A, where A: treatment with Therapeutic Hyper-CL™ lens with 5% salt solution; B: treatment with regular soft contact lens with 5% salt solution. The randomization scheme was prepared by using the SAS® random number procedure. Patients were not aware to which treatment arm they were randomized throughout the study. Group A included a 7-day treatment period with the Therapeutic Hyper-CL™ lens, 5% salt solution (1 drop 6 times a day), and Vigamox (Moxifloxacin, Alcon) eye drops (q.i.d). Group B included a 7-day treatment with a standard contact lens (PureVision, Bausch & Lomb Inc., Rochester, NY), with the same regimen of drops. There was a 7-day washout period between treatments. Patients were asked to discontinue all ophthalmic treatments for at least 7 days prior to initiation of treatment, except for steroid and antiglaucoma drops. All patients underwent a standard slit-lamp examination on days 0, 7, 14, and 21 of the study and were asked to complete a pain (grade 1–10) questionnaire at the completion of each of the two 7-day treatment periods (days 7 and 14). Central corneal thickness (CCT) was measured by optical coherence tomography (OCT). Adverse events were recorded throughout the study and infectious keratitis and allergic or toxic inflammatory reactions were predefined as related adverse effects. Patients were assessed for BCVA change, measured in early treatment diabetic retinopathy study (ETDRS) lines. BCVA testing was done during morning hours, while wearing the contact lens.

### 2.3. Therapeutic Hyper-CL™ Lens: Mechanism of Action

**Figure 1:**
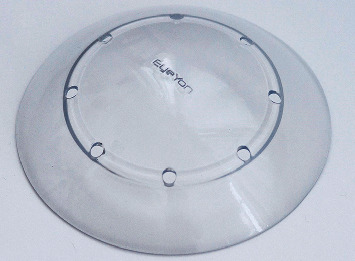
Therapeutic Hyper-CL™ lens design. The lens design consists of a dual base curve, fenestrations, and a reservoir for accumulation of therapeutic solutions.

The Therapeutic Hyper-CL™ Soft Contact Lens for Short-time Wear (up to 7 consecutive days) is a CE-marked contact lens, containing 58% water, by weight, with a *Dk*\*t* of 26. The lens is designed, with dynamic base curves, fenestrations, and reservoir (shown in Figure 1).

**Figure 2:**
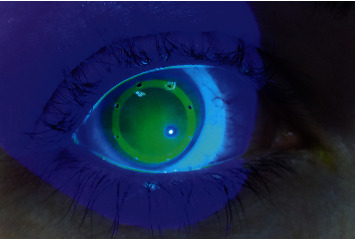
High molecular-weight fluorescein accumulation. Saline stained with high molecular weight fluorescein was applied on the Therapeutic Hyper-CL™ surface while on the patient eye. The high molecular weight fluorescein does not penetrate the lens matrix; thus, a high fluoresce pattern demonstrates the accumulation of 5% salt solution or any other therapeutic eye drops under the lens. This fluorescing pattern was shown to last on the eye for 10–20 minutes.

**Figure 3:**
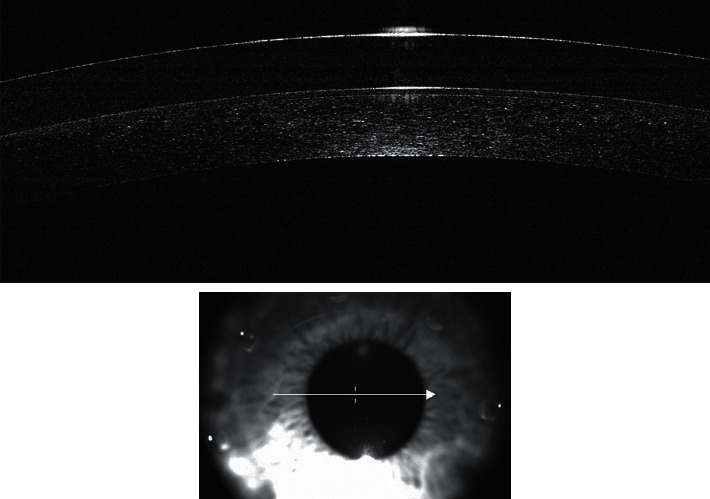
Anterior segment optical coherence tomography. The potential reservoir is demonstrated by anterior segment optical coherence tomography.

Due to the dynamic base curve of the lens, the central base curve is steeper than the peripheral, which results in a slight elevation of the lens at the center of the cornea. This elevation, forming a cavity between the lens and the cornea, creates a potential fluid reservoir. With the lens positioned mainly in the limbal area, relatively easier fit is achieved, with the reservoir enabling for retention of any applied eye drops for a prolonged period (shown in Figure 2). The fenestrations in the design of the lens allow for any instilled drops to enter the cavity, as well as for higher permeability of the lens to the diffusion of oxygen. Unpublished preclinical and in vitro studies have shown that the Therapeutic Hyper-CL™ reservoir can maintain eye drop solutions on the corneal surface for a period of at least 10 minutes, compared to 20–30 seconds without the lens (shown in Figure 3).

### 2.4. Statistical Analysis

Statistical analyses were performed using SAS V9.4 (SAS Institute, Cary NC, USA). The rates of subjects with CCT decrease and BCVA improvement were compared between the two treatments using the McNemar test. For comparison of continuous variables between the two treatments, the Paired *T*  test was used. A *P* value of ≤0.05 was considered statistically significant.

## 3. Results

Overall, 49 patients suffering from chronic corneal edema (21 males and 28 females), with an average age of 70.5 ± 11.3 years, were randomized to start either with the Therapeutic Hyper-CL™ (*n* = 24) or with control lens (*n* = 25) for 7 days. Two patients, one per treatment group, failed to complete the first week of treatment and two patients, one per treatment group, did not begin the second treatment.

### 3.1. Primary Outcome Measure

There was a significantly greater BCVA improvement rate >3 lines (30.4% versus 17.4%, *P*=0.04) and a greater proportion of patients with a >8% reduction in CCT (21.7% versus 8.9%, *P*=0.05) in the Therapeutic Hyper-CL™ group.

### 3.2. Best Corrected Visual Acuity

The mean change in BCVA lines was significantly greater for the Therapeutic Hyper-CL™ lens (3.4 ± 6.7 versus 0.9 ± 2.3, *P*=0.02). The proportion of patients with a BCVA improvement >1, 2, and 3 lines was significantly higher in the Therapeutic Hyper-CL™ group (Table 1).

### 3.3. Central Corneal Thickness

At the completion of treatment, the Therapeutic Hyper-CL™ group showed a −3.46% ± 9.71% (*P*=0.02) reduction in CCT with 21.7% demonstrating a >8% improvement in CCT. In contrast, the CCT in eyes treated with the control lens increased by +0.38 ± 8.81%, with only 8.9% demonstrating a >8% improvement in CCT (*P*=0.05).

### 3.4. Tolerability and Safety

The change in IOP (0.0 ± 3.0 mmHg versus 0.0 ± 3.0 mmHg, *P*=0.55) and pain/discomfort (− 0.1 ± 3.0 versus − 0.1 ± 2.3, *P*=0.79) was negligible and similar between groups. The most common device related adverse event was pain or discomfort which was reported similarly in both groups (8.9% versus 11.1%, *P*=0.31). There were no significant differences in both groups in terms of all other device-related adverse events (Table 2).

## 4. Discussion

This multicenter, prospective, randomized, crossover study compared the safety and efficacy of the Therapeutic Hyper-CL™ versus a standard bandage contact lens in management of corneal edema with secondary visual impairment. The proportion of patients achieving a significant improvement in CCT and BCVA was greater in the Therapeutic Hyper-CL™ group. In addition, the Therapeutic Hyper-CL™ group had a greater improvement in mean BCVA and mean CCT. Both groups had a similarly low rate of adverse events.

In attempt of avoiding low recruiting number of patients, we decided on rather promiscuous inclusion criteria regarding visual acuity and comorbidity at presentation; this led to recruitment of patients with low visual potential despite improvement in CCT. In further analysis, in order to reduce this affect when excluding patients with visual acuity <FC, the improvement in 3 lines BCVA has increased to 44%.

There are several alternatives to corneal transplantation for the management of corneal edema; however noninvasive methods comprise mostly bandage contact lenses and hypertonic saline eye drops with few clinical trials assessing the effectiveness of these modalities [11]. Luxenberg and Green [12] first reported in 1971 that 5% hypertonic saline was more effective than other hyperosmotic agents. Marisi and Aquavella [13] reported improvement in visual acuity of 60.7% of eyes with corneal edema treated with 5% saline solution for a period of three months. Knezovic et al. [10] reported that the improvement with hypertonic saline was seen mostly in patients in the early stages of bullous keratopathy. The combined use of bandage contact lenses with hypertonic saline for bullous keratopathy was first described by Gasset and Kaufman who reported improvement in a series of 49 patients [14]. Since then, bandage contact lenses have often been used for conservative treatment of corneal decompensation to relieve pain and improve visual acuity [15, 16].

The Therapeutic Hyper-CL™ lens was developed to generate a hypertonic reservoir over the corneal surface, ultimately increasing contact time of the hyperosmotic drops on the surface of the cornea. As shown, application of the Therapeutic Hyper-CL™ on edematous eyes over a 7-day treatment period brought a greater reduction in CCT compared to the control lens. As a result, a greater portion of patients benefited from a 1-, 2-, and 3-line improvement in BCVA compared to the control lens and the mean gain of vision was also significantly greater. The greater improvement in vision in the Therapeutic Hyper-CL™ group is likely due to the greater effect of the Therapeutic Hyper-CL™ in reducing CCT by reducing edema and therefore leading to a clearer cornea.

It is important to mention that the oxygen permeability (*Dk* value) of the lens is as high as a therapeutic lens and there is no need for additional support of oxygen or other materials as suggested in other papers [17].

This study has several limitations, the first of which is its relatively small sample size; however, the crossover design of the study allowed for paired analyses increasing the overall power of the study. Second, due to the differences in the design between the Therapeutic Hyper-CL™ and the bandage contact lens, it was impossible for the treating physician to be masked; however, the BCVA and CCT measurements were performed by technicians who were not aware of the treatment allocation of the examined subject and the subjects themselves were also not aware of it. Third, the relatively short follow-up time limits the ability to comment regarding potential long-term adverse events. Finally, a wide range of etiologies of corneal edema were included in this study and the limited sample size does not allow for comparison of efficacy for different etiologies. However, the Therapeutic Hyper-CL™ did show a significant advantage in terms of both CCT reduction and BCVA achieved in over 50% of patients. Future, larger, prospective studies with longer follow-up times may consider focusing on specific etiologies.

Nevertheless, the Therapeutic Hyper-CL™ lens enhanced the impact of conservative hypertonic solution therapy for corneal edema, presumably by extending hypertonic saline bioavailability and contact time with the cornea; this effect is still limited in dehydrating the cornea and as shown about 52.2% of patients improved less than 1 line or not at all; this figure has to be taken as part of the expectation when using this lens. Overall, its use significantly increased the likelihood of improvement in BCVA. The lens was comparably safe and tolerable compared to a therapeutic bandage contact lens. Patients suffering from chronic corneal edema or scheduled for corneal transplantation may benefit from this bridging treatment modality. Furthermore, patients with a poor prognosis (e.g., multiple corneal rejections, comorbidities) may particularly benefit from regular use of the Therapeutic Hyper-CL™.

## Figures and Tables

**Table 1:** Proportion of patients improving >1 line, >2 lines, and >3 lines in the Hyper-CL and bandage contact lens groups.

BCVA improvement	Hyper-CL (%)	Control (%)	*P* value
>1 line	47.8	34.8	0.04
>2 lines	43.5	28.3	0.03
>3 lines	30.4	17.4	0.04

**Table 2:** Device-related adverse events in the Hyper-CL and bandage contact lens groups.

Adverse event	Hyper-CL (%)	Control (%)	*P* value
Pain or discomfort	8.9	11.1	0.31
Conjunctival irritation	2.2	4.4	0.31
Bullae related corneal erosion	6.7	2.2	0.19
Lens intolerance	2.2	0.0	0.25

## Data Availability

The data supporting the findings of this study are available within the article; nevertheless, any additional information needed is available from the corresponding author upon reasonable request.
